# Discovery of Acupoints and Combinations with Potential to Treat Vascular Dementia: A Data Mining Analysis

**DOI:** 10.1155/2015/310591

**Published:** 2015-07-30

**Authors:** Shuwei Feng, Yulan Ren, Shilin Fan, Minyu Wang, Tianxiao Sun, Fang Zeng, Ping Li, Fanrong Liang

**Affiliations:** ^1^Chengdu University of Traditional Chinese Medicine, Chengdu, Sichuan 610075, China; ^2^Southwest Petroleum University, Chengdu, Sichuan 610500, China

## Abstract

The prevalence of vascular dementia (VaD) is high among the elderly. Acupuncture, a popular therapeutic method in China, can improve memory, orientation, calculation, and self-managing ability in VaD patients. However, in clinical acupuncture and acupuncture research, the selection of acupoints to treat VaD remains challenging. This study aimed to discover acupoints and acupoint combinations with potential for VaD based on data mining. After database searching and screening for articles on clinical trials evaluating the effects of acupuncture on VaD, 238 acupuncture prescriptions were included for further analysis. Baihui (GV 20), Sishencong (EX-HN 1), Fengchi (GB 20), Shuigou (GV 26), and Shenting (GV 24) appeared most frequently in the modern literature and are potential acupoints for VaD. Combinations between Baihui (GV 20), Sishencong (EX-HN 1), Fengchi (GB 20), Shenting (GV 24), Shuigou (GV 26), and Zusanli (ST 36) were most frequent and represent potential combinations for VaD treatment. These results provide a reference for the selection and combination of acupoints to treat VaD in clinical acupuncture and acupuncture research.

## 1. Introduction

The selection of acupoints plays a critical role in the therapeutic effects of acupuncture. However, the selection of proper acupoints remains challenging, contributing to the limited therapeutic effects and application of acupuncture. Data mining has been used to discover potential acupoints from the expansive relevant literature. This method has been used to suggest acupoints on the Shaoyang Meridian for migraine treatment based on their high frequency in the literature [1]. Based on the results of data mining, a subsequent clinical trial confirmed that acupuncture was effective for the treatment of migraine and that acupoints on the Shaoyang Meridian were more effective than acupoints on other meridians [2]. Data mining has also been used to discover potential Chinese herbs for the effective treatment of specific diseases [3, 4]. These results support data mining as a promising method to discover acupoints with potential for treating diseases.

Vascular dementia (VaD) refers to cognitive impairment caused by changes in the blood circulation of the brain [5]. Its clinical manifestations include confusion or short-term memory problems, wandering, getting lost in familiar places, walking with rapid and shuffling steps, losing bladder or bowel control, laughing or crying inappropriately, difficulty in following instructions, and problem with counting money and conducting monetary transactions. At the late stage, VaD patients may have severe impairment of basic activities of daily living and lack the capacity to make appropriate decisions regarding their choices and preferences [6]. A recent population-based survey reported that the prevalence of VaD among individuals aged 65 years and older was 1.5% [7]. It has been predicted that dementia will affect 80 million people worldwide by 2040 [8]. The annual cost of care per patient is estimated to be US$17,000–55,200 for severe dementia, placing a heavy economic burden on families and society [9].

Acupuncture, a primary therapeutic method in traditional Chinese medicine (TCM), can improve memory, orientation, calculation, and self-managing ability in VaD patients [10–12]. The therapeutic effects of acupuncture are achieved via multiple pathways, including antioxidative effects, antiapoptotic effects, and neurotrophic effects [11, 13–15]. However, acupoint selection remains a challenge in the use of acupuncture to treat VaD. According to our preliminary statistics, more than 100 acupoints distributed in 13 meridians have been recorded in the modern literature for the treatment of VaD. The most effective acupoints for the treatment of VaD and the selection of acupoints for combination remain to be elucidated, representing a major limitation for clinical therapeutic effects and the application of acupuncture for VaD.

To shed some light on the selection of acupoints and acupoint combinations to treat VaD in clinical acupuncture and acupuncture research, this study aimed to discover acupoints and acupoint combinations that have potential to treat VaD via data mining.

## 2. Materials and Methods

**Figure 1:**
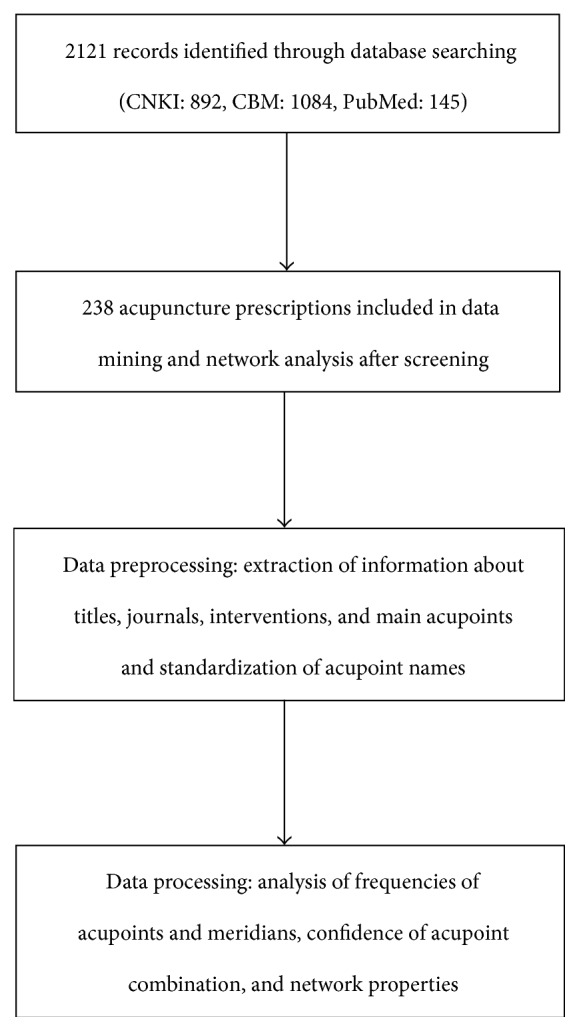
Flow of information through the different phases of data mining.

The flow of information through the various phases of data mining is illustrated in Figure 1.

### 2.1. Inclusion Criteria for Considering Acupoint Prescriptions for Data Mining

#### 2.1.1. Types of Studies

Clinical trials evaluating the effect of TCM acupuncture with or without randomization methods were included. Trials with or without controls were also included. The control interventions included no treatment, sham acupuncture, Western medicine, TCM herbs, nontraditional acupuncture, and TCM acupuncture containing another acupoint prescription which is different from the one in observation group. Language was restricted to Chinese and English.

#### 2.1.2. Types of Participants

Clinical trials involving adult participants diagnosed with VaD were included.

#### 2.1.3. Types of Interventions

Clinical trials evaluating TCM acupuncture were included. Acupuncture can be used alone or in combination with other types of interventions. TCM acupuncture involves inserting needles into traditional meridian acupoints and extraordinary acupoints. Electrical stimulation of the needles may be used. Trials using moxibustion alone or as a cointervention with acupuncture were also included.

#### 2.1.4. Effectiveness of Acupoint Prescriptions

Acupoint prescriptions for the disease and not particular syndromes of VaD were included. There should be statistical differences in symptoms between before and after acupuncture. In a controlled trial, patients treated with acupuncture alone or in combination should receive greater benefit than patients who do not receive acupuncture therapy. The control interventions included no treatment, sham acupuncture, Western medicine, TCM herbs, nontraditional acupuncture, and TCM acupuncture containing another acupoint prescription which is different from the one in observation group. If the studies compared the therapeutic effects of different acupoint prescriptions, the most effective acupoint prescription was included.

### 2.2. Exclusion Criteria

#### 2.2.1. Types of Studies

Case reports, reviews, systematic reviews, and meta-analyses were excluded.

#### 2.2.2. Types of Participants

Trials evaluating the therapeutic effect of acupuncture for Alzheimer's disease, traumatic dementia, and other subtypes of dementia were excluded. Studies on animals were also excluded.

#### 2.2.3. Types of Interventions

Trials stimulating Ashi points alone were excluded. Trials of dry needling or trigger point therapy, therapies that are based on principles of western anatomy and physiology, were excluded. Trials of laser acupuncture and noninvasive electrostimulation (e.g., using electrodes on the skin rather than needles or moxibustion to stimulate the acupoints) were excluded to limit the focus to TCM acupuncture. Trials evaluating acupressure, a form of massage, were excluded as well. Finally, trials of micropuncture were excluded because micropuncture is a nontraditional acupuncture practice that is based on the principle that the head (or ear, nose, eye, abdomen, ankle, etc.) is a microsystem of the entire body.

#### 2.2.4. Effectiveness of Acupoint Prescriptions

Acupoint prescriptions for a particular syndrome of VaD were excluded. Acupoint prescriptions with no statistical improvement of symptoms were also excluded. When the therapeutic effects of different acupoint prescriptions were compared in a study, all acupoint prescriptions except the most effective one were excluded.

### 2.3. Searching Methods for Identification of Studies

#### 2.3.1. Data Sources

PubMed (http://www.pubmed.com (1966 to 2012)), the Chinese BioMedicine Database (CBM) (http://www.sinomed.ac.cn (1978 to 2012)), and China National Knowledge Infrastructure (CNKI) (http://www.cnki.net (1912 to 2012)) were searched for modern literature on acupuncture treatment for VaD.

#### 2.3.2. Searching Strategy

The searching strategy used the following key words: (I) “acupuncture” OR “electroacupuncture” OR “moxibustion” OR “meridian” OR “acupoint”; (II) “dementia” OR “vascular dementia” OR “Alzheimer's disease.” The searching strategy included literature on acupuncture treatment for Alzheimer's disease (AD) because the modern literature on acupuncture for VaD overlaps with that on AD.

### 2.4. Data Collection

Two reviewers independently screened the title and abstract of every record retrieved from the literature searches. All potentially relevant articles were investigated as full text in English or Chinese. In cases of disagreement, a trial was included or excluded based on discussion between the two reviewers or after a third reviewer reviewed the information. For duplicate publications, the final publication was used.

### 2.5. Data Preprocessing

Information about titles, journals, interventions, and main acupoints was extracted using the self-established Data Excavation Platform of Acupoint Specificity for data mining. Because acupoints have aliases, the names of acupoints were standardized according to* Fundamentals of Acupuncture* [16].

### 2.6. Data Processing

#### 2.6.1. Frequencies of Acupoints

The frequencies of acupoints, meridians, and acupoints on different body parts were analyzed using the Data Excavation Platform of Acupoint Specificity.

#### 2.6.2. Association Rules Mining

Apriori Algorithm for association rules mining [17] was adopted to analyze the frequencies and support of acupoint combinations. According to the definition of association rules mining [18], the following can be a statement of association rules mining for acupoint combination. Let *I* = {*i*_1_, *i*_2_,…, *i*_*m*_} be a set of acupoints. Let *D* be a set of acupoint prescriptions, where each acupoint prescription *T* is a set of acupoints such that *T*⊆*I*. Associated with each acupoint prescription is a unique identifier, called TID. An acupoint prescription *T* contains *X*, a set of some acupoints in *I*, if *X*⊆*T*. The rule *X*-*Y* has* support s* in the acupoint prescription set *D* if *s*% of acupoint prescriptions in *D* contain *X* ∪ *Y*.

#### 2.6.3. Measurement of Network Properties

Community structure is a common characteristic of complex networks and is characterized by more dense internal connections within groups of nodes than with the rest of the network. In this study, a hierarchical agglomeration was adopted to detect community structure according to Clauset et al. [19]. We also focused on investigating the set of the most influential nodes in acupoint networks of VaD, defined as the nodes with the highest *k*-core value [20]. The *k*-core method is predominantly used in analyzing social networks. We employed the *k*-core method to obtain the cores of different acupoints. The *k*-core method was implemented as follows. First, all 1-degree nodes were removed, and the nodes were further pruned until no 1-degree nodes remained. The remaining nodes formed the 2-core node set. The pruning process was repeated in a similar manner for other nodes in the network assigned to the corresponding cores (denoted by *k*s). The nodes with the largest *k*-core value were defined as the network core. The degree of each acupoint was also analyzed to measure the involvement of the node in the network. The degree refers to the number of nodes to which a focal node is connected [21]. Betweenness centrality was also used to analyze an acupoint's centrality in the network. Centrality is an important concept for the analysis of networks, and betweenness centrality is one of the most prominent measures of centrality. It is used to measure the degree to which a node is in a position of brokerage by summing up the fractions of shortest paths between other pairs of vertices that pass through it [22].

## 3. Results

### 3.1. Overall Profile of Acupuncture Prescriptions

**Figure 2:**
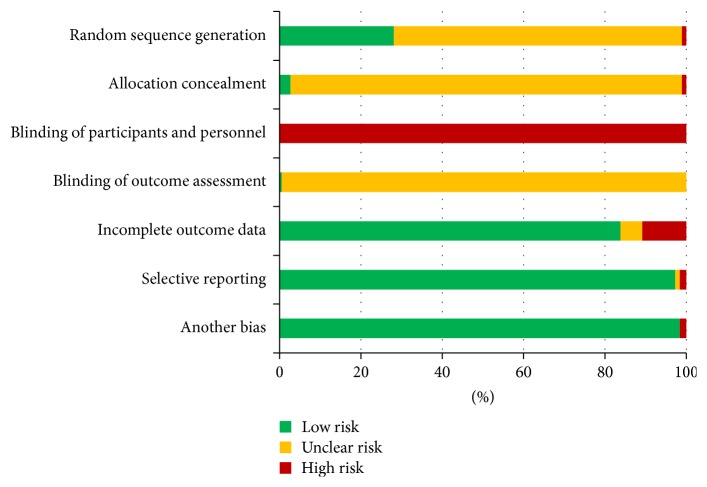
Whole view on the study quality of the 185 CCTs.

Database searching identified 892 records in CNKI, 1084 records in CBM, and 145 records in PubMed. After screening, 238 acupuncture prescriptions in 238 articles were included. Among the 238 trials, 185 are controlled clinical trials (CCTs), while the other 53 trials have no controls. The whole view on the study quality of the 185 CCTs were shown in Figure 2.

### 3.2. Frequencies of Acupoints and Meridians

**Figure 3:**
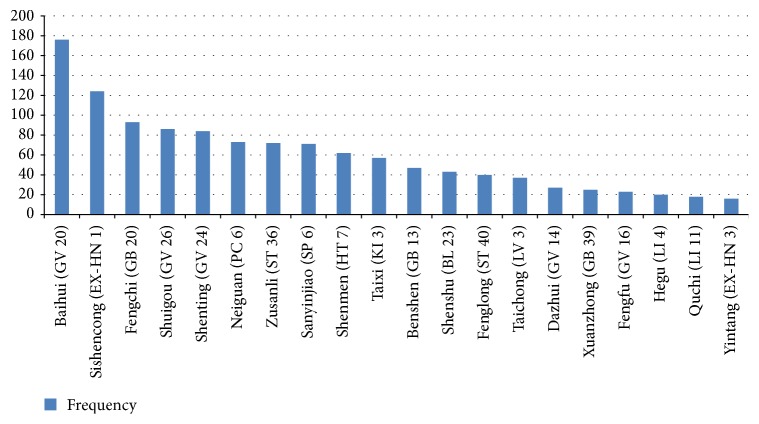
The 20 most frequent acupoints and their frequencies.

Approximately 109 meridian-acupoints distributed over 13 meridians and 7 extraordinary acupoints have been recorded for 1400 and 133 times, respectively, in modern literature on acupuncture treatment for VaD. The most frequently used meridian was the Governor Meridian (477 times). Other frequently used meridians included the Gallbladder Meridian of Foot Shaoyang and the Stomach Meridian of Foot Yangming, which were reported for 218 and 124 times, respectively. Extraordinary acupoints were also frequently used. Baihui (GV 20), Sishencong (EX-HN 1), Fengchi (GB 20), Shuigou (GV 26), and Shenting (GV 24), which were among the top five acupoints in frequency, were recorded for 176, 124, 93, 86, and 84 times, respectively. The frequencies of each meridian and acupoint are shown in Table 1. The twenty most frequently used acupoints are shown in Figure 3.

### 3.3. Frequencies of Acupoints on Different Body Parts

**Figure 4:**
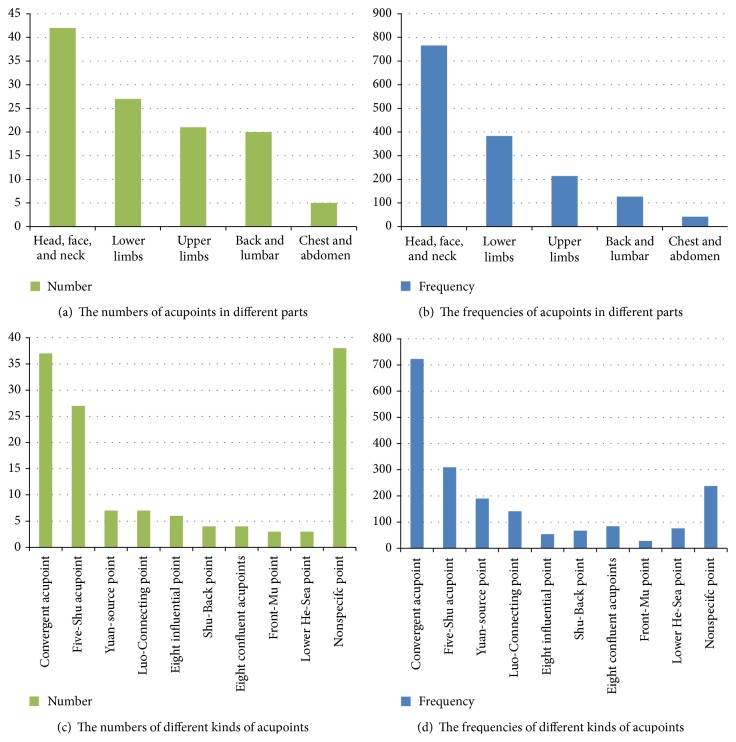
The frequencies and numbers of acupoints in different body parts and different types of acupoints.

Acupoints on the head, face, and neck were used most frequently, with a total number of 42 acupoints and a total frequency of 766 times, followed by acupoints on the lower limbs (383 times), upper limbs (214 times), back and lumbar (127 times), and chest and abdomen (43 times) (Figures 4(a) and 4(b)).

### 3.4. Frequencies of Specific Acupoints

Specific acupoints represented 78 of the 116 acupoints (67.24%). Specific acupoints have been used 1292 times, representing 84.28% of the total frequency of all acupoints (Figures 4(c) and 4(d)).

### 3.5. Frequencies and Support of Acupoint Combinations

Acupoint combinations between Baihui (GV 20), Sishencong (EX-HN 1), Fengchi (GB 20), Shenting (GV 24), Shuigou (GV 26), and Zusanli (ST 36) were used most frequently. The 15 most frequently used acupoint combinations and their support and confidence are shown in Table 2.

### 3.6. Community Structure

**Figure 5:**
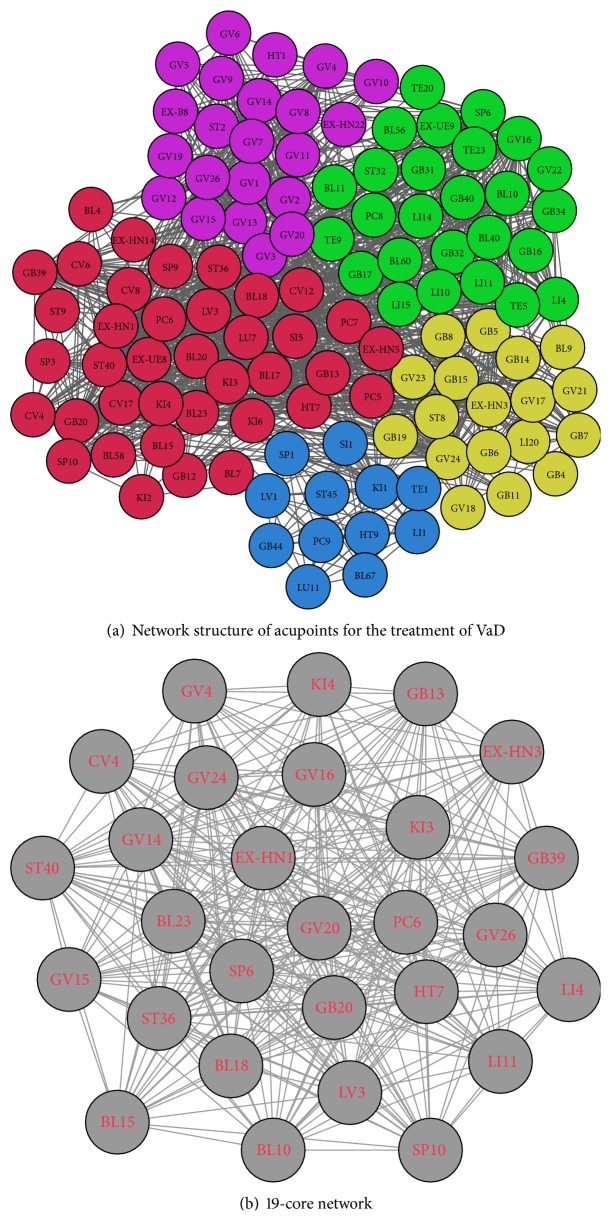
(a) Network structure of acupoints for the treatment of VaD. Blue nodes (Community A) are all Jing-Well acupoints. Yellow nodes (Community B) are all acupoints on the face and head. Most green nodes (Community C) are acupoints on four limbs. Most purple nodes (Community D) are acupoints belonging to Governor Vessel. Most red nodes (Community E) are specific acupoints or acupoints with specific therapeutic effects, and only this community contains Bach-Shu acupoints and acupoints on the abdomen. Acupoints within the same community are more densely connected with each other than acupoints from different communities. (b) 19-core network. There are 28 acupoints in the 19-core network. They are core acupoints in the treatment of VaD.

Community detection resulted in the division of the 116 acupoints into 5 communities. Nodes of the same color belong to the same community. The community structure is shown in Figure 5(a).

### 3.7. Acupoint *K*-Core Network

The largest *k*-core value was 19. At this value, there were 28 nodes, corresponding to Hegu (LI 4), Quchi (LI 11), Zusanli (ST 36), Fenglong (ST 40), Sanyinjiao (SP 6), Xuehai (SP 10), Shenmen (HT 7), Tianzhu (BL 10), Xinshu (BL 15), Ganshu (BL 18), Shenshu (BL 23), Taixi (KI 3), Dazhong (KI 4), Neiguan (PC 6), Benshen (GB 13), Fengchi (GB 20), Xuanzhong (GB 39), Taichong (LV 3), Mingmen (GV 4), Dazhui (GV 14), Yamen (GV 15), Fengfu (GV 16), Baihui (GV 20), Shenting (GV 24), Shuigou (GV 26), Guanyuan (CV 4), Sishencong (EX-HN 1), and Yintang (EX-HN 3), as shown in Figure 5(b).

### 3.8. Degree

**Figure 6:**
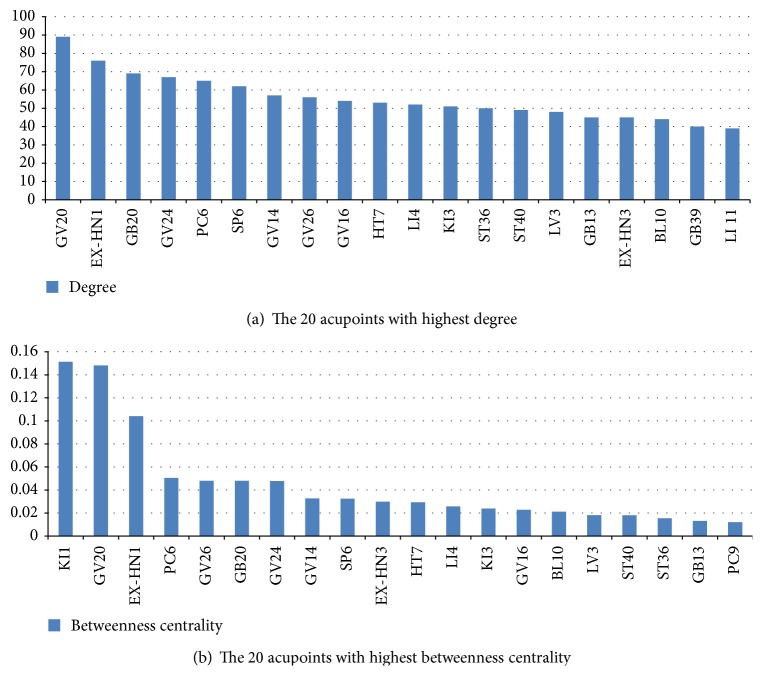
The 20 acupoints with highest degree and the 20 acupoints with highest betweenness centrality.

The top 20 acupoints in degree are shown in Figure 6(a). Baihui (GV 20), Sishencong (EX-HN 1), Fengchi (GB 20), Shenting (GV 24), and Neiguan (PC 6) had the highest degrees, with values of 89, 76, 68, 67, and 65, respectively.

### 3.9. Betweenness Centrality

The top 20 acupoints in betweenness centrality are shown in Figure 6(b). Yongquan (KI 1), Baihui (GV 20), and Sishencong (EX-HN 1) had the highest betweenness centrality.

## 4. Discussion

### 4.1. Potential Acupoints and Combinations for VaD

In this study, acupoints and combinations with potential for treating VaD were discovered. These results may provide some reference for the selection of acupoints in treatment for VaD, which may promote the therapeutic effects in clinical practice. The results suggest that Baihui (GV 20), Sishencong (EX-HN 1), Fengchi (GB 20), Shuigou (GV 26), and Shenting (GV 24) are potential acupoints for treating VaD. In terms of meridian, acupoints on the Governor Meridian have potential for treating VaD. From the perspective of combinations, combinations between such acupoints as Baihui (GV 20), Sishencong (EX-HN 1), Fengchi (GB 20), Shenting (GV 24), Shuigou (GV 26), and Zusanli (ST 36) have potential for treating VaD. In addition, acupoints on the head, face, and neck have more potential for VaD than acupoints on other regions of the body. Specific acupoints have more potential than nonspecific acupoints. Specific acupoints, with specific names, are a group of acupoints on fourteen meridians with specific therapeutic effects. There are ten types of specific acupoints, Five-Shu acupoints, Yuan-Primary acupoints, Luo-Connecting acupoints, Xi-Cleft acupoints, Lower He-Sea acupoints, Back-Shu acupoints, Front-Mu acupoints, eight influential acupoints, eight confluent acupoints connecting the eight extra meridians, and convergent acupoints.

Community detection divided the acupoints into 5 communities. Acupoints within the same community have some characteristics in common. Blue nodes (Community A) were all Jing-Well acupoints. Yellow nodes (Community B) were all acupoints on the face and head. Most green nodes (Community C) were acupoints on the four limbs. Most purple nodes (Community D) were acupoints belonging to Governor Vessel. Most red nodes (Community E) were specific acupoints or acupoints with specific therapeutic effects, and only this community contained Bach-Shu acupoints and acupoints on the abdomen. Acupoints within the same community were more densely connected with each other compared with acupoints from different communities, indicating that an acupoint was more often used with acupoints within the same community compared with acupoints within other communities.

The 19-core network indicated that 28 acupoints, including Hegu (LI 4), Quchi (LI 11), Zusanli (ST 36), Fenglong (ST 40), Sanyinjiao (SP 6), Xuehai (SP 10), Shenmen (HT 7), Tianzhu (BL 10), Xinshu (BL 15), Ganshu (BL 18), Shenshu (BL 23), Taixi (KI 3), Dazhong (KI 4), Neiguan (PC 6), Benshen (GB 13), Fengchi (GB 20), Xuanzhong (GB 39), Taichong (LV 3), Mingmen (GV 4), Dazhui (GV 14), Yamen (GV 15), Fengfu (GV 16), Baihui (GV 20), Shenting (GV 24), Shuigou (GV 26), Guanyuan (CV 4), Sishencong (EX-HN 1), and Yintang (EX-HN 3) are core acupoints in the network.

Baihui (GV 20), Sishencong (EX-HN 1), Fengchi (GB 20), Shenting (GV 24), and Neiguan (PC 6) had the highest degrees. This result indicates that these 5 acupoints have been combined with more acupoints than other acupoints. These acupoints have specific therapeutic effects on VaD. Therefore, these acupoints can be used together with other acupoints to enhance therapeutic effects.

Yongquan (KI 1), Baihui (GV 20), Sishencong (EX-HN 1), Neiguan (PC 6), and Shenting (GV 24), which belonged to 4 different communities, had higher betweenness centrality. Yongquan (KI 1), which had the highest betweenness centrality, did not have a relatively high degree. However, it connects Jing-Well acupoints with other types of acupoints, resulting in a high betweenness centrality. Acupoints with higher betweenness centrality play an important role in connecting different types of acupoints. Jing-Well acupoints, except Yongquan (KI 1), were often used with other Jing-Well acupoints only. Yongquan (KI 1) was not only used with other Jing-Well acupoints but also with other types of acupoints, such as other types of specific acupoints, nonspecific acupoints, and acupoints on other parts. The high betweenness centrality suggested that Yongquan (KI 1) may have multiple effects compared with other Jing-Well acupoints in treatment of VaD. From the perspective of TCM theory, Jing-Well acupoints can restore consciousness. Yongquan (KI 1) was also used to tonify kidney in treatment of VaD. Therefore, Yongquan (KI 1) is used not only with other Jing-Well acupoints but also with other types of acupoints, such as other types of specific acupoints, nonspecific acupoints, and acupoints on other parts.

### 4.2. Underlying Molecular Mechanism of the Acupoint with the Most Potential to Treat VaD

According to our results, Baihui (GV 20), which had the highest frequency, has the most potential to treat VaD. A systematic review and meta-analysis also suggested that Baihui (GV 20) is a principal acupoint for acute intracerebral hemorrhage (ICH); in animal models of acute ICH, there was no difference in efficacy between Baihui (GV 20) alone and Baihui (GV 20) plus other acupoints [23].

Molecular biology studies have provided insights into the mechanisms underlying the effects of Baihui (GV 20) in VaD treatment, including antioxidant effects, antiapoptotic effects, neurotrophic effects, reduced blood-brain barrier (BBB) permeability, and regulation of the cholinergic and dopaminergic systems. Acupuncture at Baihui (GV 20) in combination with other acupoints decreases levels of 8-hydroxy-2′-deoxyguanosine, a product of oxidative damage to DNA induced by free radicals, suggesting that the benefit of acupuncture is partly due to antioxidant effects [11]. Acupuncture exerts therapeutic effects on VaD via antiapoptosis. The tumor suppressors p53 and Noxa are important in regulating apoptosis and mediate hypoxic cell death [24, 25]. Electroacupuncture at Baihui (GV 20), Dazhui (GV 14), and Shenshu (BL 23) blocks expression of p53 and Noxa in the hippocampal CA1 region of VaD rats [26]. Acupuncture at Baihui (GV 20) can improve neurogenesis via regulating brain-derived neurotrophic factor (BDNF) and cyclic AMP response element-binding protein (CREB). BDNF, which is essential for synaptic plasticity and is coupled to CREB activation [27], is important for long-term memory storage [28]. CREB is required for the proliferation, growth, survival, and differentiation of all types of cells. In the brain, the CREB and CRE-mediated system is involved in memory, learning, synaptic transmission, neuron survival, differentiation, and axon growth [29]. Acupuncture at Baihui (GV 20) significantly increases the levels of BDNF [15, 30], CREB proteins, CREB mRNA [30], and phosphorylated CREB, the active form of CREB [15]. The molecular mechanism underlying acupuncture at Baihui (GV 20) also involves cholinergic system regulation. Decreased cholinergic function in the brain can result in a decline in memory and cognitive function [31]. Acupuncture at Baihui (GV 20) significantly increases the levels of choline acetyltransferase (ChAT) and restores the expression of choline transporter 1 (CHT1) and vesicular acetylcholine transporter (VAChT) [30]. The dopaminergic system is also involved in the mechanism underlying the treatment of VaD with acupuncture at Baihui (GV 20). Dopamine is a key regulator in specific synaptic changes observed at certain stages of learning and memory and of synaptic plasticity [32]. Acupuncture at Baihui (GV 20) increases dopamine levels in chronic cerebral hypoperfusion and ischemia-reperfusion injured rats [33]. In addition, acupuncture at Baihui (GV 20) and Zusanli (ST 36) preserves the integrity of the BBB, reducing BBB permeability. The BBB is constructed of tight conjunctions, including occludin and claudin-5, which form the endothelial barrier. Reduced expression of ZO-1, claudin-5, and occludin mRNA and protein contributes to BBB breakdown and edema in the ischemic brain [34]. Electroacupuncture at Baihui (GV 20) and Zusanli (ST 36) reduces brain damage and related behavioral deficits via upregulation of tight conjunction proteins, including ZO-1, claudin-5, and occludin [35]. These findings reveal parts of the molecular mechanism underlying acupuncture at Baihui (GV 20) to treat VaD.

### 4.3. Acupoints Selection in Treatment for VaD

The proper selection of acupoints is essential for the therapeutic effects of acupuncture because acupoints are specific with regard to morphological structure, biophysical properties, pathological response, and stimulating effects [36]. This specificity differentiates acupoints from nonacupoints as well as different acupoints from one another. The specific therapeutic effects of different acupoints have been reported for migraine [37], functional dyspepsia [38], ischemic stroke [39], and so forth.

The specificity of acupoints for the treatment of VaD has also been reported. Phosphorylated CREB levels were significantly increased after acupuncture therapy of needling Baihui (GV 20) and Zusanli (ST 36) compared to sham acupuncture therapy of needling nonacupoints [15]. Baihui (GV 20), Shuigou (GV 26), and Shenmen (HT 7) are all among the 10 acupoints with the most potential. A clinical trial demonstrated that needling Baihui (GV 20), Shuigou (GV 26), and Shenmen (HT 7) were all effective in improving the symptoms of VaD. However, their therapeutic effects differ. Needling Baihui (GV 20) improved calculation ability and short-term memory and corrected the personality changes of VaD patients, while needling Shuigou (GV 26) improved naming ability and short-term memory. The therapeutic effects of needling Baihui (GV 20) and Shuigou (GV 26) were superior to those of needling Shenmen (HT 7) [40]. A PET and SPECT study revealed that needling these three different acupoints in VaD patients affected different brain areas. Needling Baihui (GV 20) activated the inner temporal system, the thalamencephalon system, and the prefrontal cortical system. Needling Shuigou (GV 26) activated the prefrontal cortical system. Needling Shenmen (HT 7) generated an effect similar to but weaker than the effect generated by needling Shuigou (GV 26) [41]. These findings demonstrate that different acupoints have different therapeutic effects in acupuncture treatment for VaD. Consequently, the selection of acupoints, which directly influences the therapeutic effects of acupuncture, should be considered carefully. According to our results based on data mining, Baihui (GV 20), Sishencong (EX-HN 1), Fengchi (GB 20), Shuigou (GV 26), and Shenting (GV 24), which have higher frequencies in the modern literature, may have better therapeutic effects on VaD.

### 4.4. Single Acupoint or Acupoint Combination

Acupoint combinations also influence the therapeutic effects of acupuncture. An acupoint combination is considered to have a synergistic effect that enhances the therapeutic effect of acupuncture. For example, a lower prevalence of postoperative nausea and vomiting in patients treated with Neiguan (PC 6) plus Hegu (LI 4) was observed compared with those treated with Neiguan (PC 6) only [42]. In spite of extensive evidence suggesting a synergistic effect of acupoint combination and supporting its use, some studies have reported antagonistic effects [43–45]. An antagonistic effect occurs when one acupoint weakens the therapeutic effect of another acupoint [46]. For example, electroacupuncture can improve gastrointestinal movement in rats. The effect of needling Pishu (BL 20) alone was better than the effect of needling Pishu (BL 20) and Zusanli (ST 36) at the same time [44]. Therefore, whether the effect of acupoint combination is better than a single acupoint still remains a question and needs to be further studied.

Some studies have compared single acupoints and an acupoint combination for the treatment of VaD. The therapeutic effect of needling Baihui (GV 20), Shuigou (GV 26), and Shenmen (HT 7) in combination was better than the effects obtained by needling each alone [40]. In addition, needling Baihui (GV 20), Shuigou (GV 26), and Shenmen (HT 7) simultaneously activated more brain areas related to intellectual activities compared with needling each alone, generating a more extensive effect on the brain [41]. Antagonistic effects in acupuncture therapy for VaD have not been reported but may occur. Most acupuncture prescriptions for VaD contain acupoint combinations, and the use of acupoint combinations is supported. However, acupoint combinations should be selected carefully to avoid antagonistic effects. It is hard to tell whether a combination of acupoints will exert antagonistic effects with current knowledge or TCM theory. As abovementioned, acupoints with similar functions can exert antagonistic effects. There are many acupoints, and the number of acupoint combinations will grow geometrically. To test the antagonistic effects of each combination one by one is an exhausting job. To avoid antagonistic effects as possible, the acupoint prescriptions should be simplified as possible. The general principle is to select acupoints with relatively better therapeutic effects and acupoints with multiple indications and not to select many acupoints.

### 4.5. Limitations

This study has limitations as follows. First, other factors that influence acupuncture, such as manipulation and treatment duration, were not analyzed in this study. These data can be further mined in future studies. Second, largely due to the lack of treatment based on syndrome differentiation and different methods of syndrome differentiation in modern literature, potential acupoints and combinations for different syndromes of VaD were not analyzed. Although treatment based on syndrome differentiation is important and is often emphasized in TCM, treatment based on disease differentiation is equally important. Third, the real therapeutic effects of acupoints and combinations on VaD cannot be reflected by frequencies in the literature. However, these results suggest some potential acupoints and combinations to be explored in future clinical trials to validate the effects of acupuncture on VaD. To optimize the acupoint prescription, data may be extracted not only from literature, but also from clinical practice. Further research can collect acupoint prescriptions and symptom improvements in clinical practice and optimize acupoint prescription with data mining. Mining clinical data has been practiced to optimize prescription of Chinese herbal medicine for unstable angina by Feng et al. [47]. In their study, five main symptoms of patients with unstable angina, the severity of each symptom, and the prescription of each patient were collected. The levels of average discounted reward (ADR) of different prescriptions were calculated to evaluate the clinical efficacy of different treatment options, with some optimized prescriptions achieved. Future studies can mine clinical data to optimize acupoint prescription.

## 5. Conclusions

In this study, data mining was used to discover potential acupoints and combinations for VaD. Potential acupoints include Baihui (GV 20), Sishencong (EX-HN 1), Fengchi (GB 20), Shuigou (GV 26), and Shenting (GV 24). In addition, combinations between Baihui (GV 20), Sishencong (EX-HN 1), Fengchi (GB 20), Shenting (GV 24), Shuigou (GV 26), and Zusanli (ST 36) were potential combinations. Based on our results, Baihui (GV 20) and Sishencong (EX-HN 1) should be selected with priority. Acupoints in head, compared to acupoints in other parts, should also be selected with priority.

## Figures and Tables

**Table 1:** Statistics of meridians and acupoints in the modern literature on acupuncture treatment for VaD.

Number	Meridian	Frequency	Number of acupoints	Acupoints and their frequencies
1	GV	477	26	Baihui (GV 20) 176, Shuigou (GV 26) 86, Shenting (GV 24) 84, Dazhui (GV 14) 27, Fengfu (GV 16) 23, Yintang (EX-HN 3) 16, Naohu (GV 17) 13, Shangxing (GV 23) 11, Mingmen (GV 4) 8, Yamen (GV 15) 5, Qiangjian (GV 18) 5, Qianding (GV 21) 4, Yaoyangguan (GV 3) 3, Zhiyang (GV 9) 3, Jinsuo (GV 8) 2, Shendao (GV 11) 1, Zhongshu (GV 7) 1, Taodao (GV 13) 1, Lingtai (GV 10) 1, Changqiang (GV 1) 1, Xuanshu (GV 5) 1, Yaoshu (GV 2) 1, Shenzhu (GV 12) 1, Jizhong (GV 6) 1, Houding (GV 19) 1, Xinhui (GV 22) 1

2	GB	218	20	Fengchi (GB 20) 93, Benshen (GB 13) 47, Xuanzhong (GB 39) 24, Shuaigu (GB 8) 7, Wangu (GB 12) 6, Toulinqi (GB 15) 6, Qubin (GB 7) 5, Xuanli (GB 6) 4, Naokong (GB 19) 4, Zuqiaoyin (GB 44) 3, Yanglingquan (GB 34) 3, Yangbai (GB 14) 3, Hanyan (GB 4) 2, Qiuxu (GB 40) 2, Fengshi (GB 31) 2, Touqiaoyin (GB 11) 2, Xuanlu (GB 5) 2, Muchuang (GB 16) 1, Zhengying (GB 17) 1, Zhongdu (GB 32) 1

3	EX-HN	133	7	Sishencong (EX-HN 1) 123, Taiyang (EX-HN 5) 4, Wailaogong (EX-UE 8) 2, Shiqizhui (EX-B 8) 1, Baxie (EX-UE 9) 1, Anmian (EX-HN 22) 1, Yiming (EX-HN 14) 1

4	ST	124	7	Zusanli (ST 36) 72, Fenglong (ST 40) 40, Touwei (ST 8) 6, Lidui (ST 45) 2, Sibai (ST 2) 2, Futu (ST 32) 1, Renying (ST 9) 1

5	BL	103	15	Shenshu (BL 23) 43, Ganshu (BL 18) 12, Tianzhu (BL 10) 10, Pishu (BL 20) 8, Feiyang (BL 58) 8, Geshu (BL 17) 7, Xinshu (BL 15) 4, Zhiyin (BL 67) 2, Kunlun (BL 60) 2, Yuzhen (BL 9) 2, Tongtian (BL 7) 1, Chengjin (BL 56) 1, Dazhu (BL 11) 1, Weizhong (BL 40) 1, Qucha (BL 4) 1

6	SP	96	5	Sanyinjiao (SP 6) 71, Xuehai (SP 10) 14, Taibai (SP 3) 8, Yinbai (SP 1) 2, Yinlingquan (SP 9) 1

7	PC	86	5	Neiguan (PC 6) 73, Zhongchong (PC 9) 4, Daling (PC 7) 4, Jianshi (PC 5) 3, Laogong (PC 8) 2

8	KI	83	5	Taixi (KI 3) 57, Dazhong (KI 4) 12, Yongquan (KI 1) 10, Zhaohai (KI 6) 8, Rangu (KI 2) 2

9	HT	67	3	Shenmen (HT 7) 62, Shaochong (HT 9) 3, Jiquan (HT 1) 2

10	LI	45	7	Hegu (LI 4) 20, Quchi (LI 11) 18, Shangyang (LI 1) 2, Shousanli (LI 10) 2, Binao (LI 14) 1, Jianyu (LI 15) 1, Yingxiang (LI 20) 1

11	CV	43	5	Qihai (CV 6) 11, Zhongwan (CV 12) 10, Guanyuan (CV 4) 9, Danzhong (CV 17) 9, Shenque (CV 8) 4

12	LV	40	2	Taichong (LV 3) 37, Dadun (LV 1) 3

13	TE	11	5	Waiguan (TE 5) 6, Guanchong (TE 1) 2, Sizhukong (TE 23) 1, Jiaosun (TE 20) 1, Sidu (TE 9) 1

14	LU	4	2	Shaoshang (LU 11) 3, Lieque (LU 7) 1

15	SI	3	2	Shaoze (SI 1) 2, Yanggu (SI 5) 1

16	Total	1,533	116	

GV, Governor Meridian; GB, Gallbladder Meridian of Foot Shaoyang; EX-HN, extraordinary acupoint; ST, Stomach Meridian of Foot Yangming; BL, Bladder Meridian of Foot Taiyang; SP, Spleen Meridian of Foot Taiyin; PC, Pericardium Meridian of Hand Jueyin; KI, Kidney Meridian of Foot Shaoyin; HT, Heart Meridian of Hand Shaoyin; CV, Conception Vessel; LI, Large Intestine Meridian of Hand Yangming; LV, Liver Meridian of Foot Jueyin; TE, Triple Energizer of Hand Shaoyang; LU, Lung Meridian of Hand Taiyin; SI, Small Intestine of Hand Taiyang. Frequencies of meridians refer to the total frequencies of acupoints on the same meridian. Number of acupoints refer to the total number of acupoints on the same meridian.

**Table 2:** Statistics of the 15 most frequently used acupoint combinations in the treatment of VaD.

Number	Acupoint combination	Frequency	Support (%)
1	Baihui (GV 20), Sishencong (EX-HN 1)	98	41.18
2	Baihui (GV 20), Fengchi (GB 20)	81	34.03
3	Baihui (GV 20), Shuigou (GV 26)	72	29.83
4	Baihui (GV 20), Shenting (GV 24)	70	29.41
5	Baihui (GV 20), Zusanli (ST 36)	62	26.05
6	Sishencong (EX-HN 1), Fengchi (GB 20)	60	25.21
7	Baihui (GV 20), Sanyinjiao (SP 6)	57	23.95
8	Baihui (GV 20), Neiguan (PC 6)	54	22.69
9	Sishencong (EX-HN 1), Shuigou (GV 26)	51	21.43
10	Sishencong (EX-HN 1), Baihui (GV 20), Fengchi (GB 20)	51	21.43
11	Baihui (GV 20), Shenmen (HT 7)	51	21.43
12	Sishencong (EX-HN 1), Shenting (GV 24)	49	20.59
13	Sishencong (EX-HN 1), Neiguan (PC 6)	46	19.33
14	Baihui (GV 20), Taixi (KI 3)	46	19.33
15	Sishencong (EX-HN 1), Baihui (GV 20), Shuigou (GV 26)	43	18.07

Support refers to the percentage of acupoint prescriptions containing the acupoint combination.

## References

[1] Chen Q., Wu X., Lu S. F. (2009). Meridian acupoints selection in acupuncture treatment for migraine: the main characters and relevant factors analysis. *Liaoning Journal of Traditional Chinese Medicine*.

[2] Li Y., Zheng H., Witt C. M. (2012). Acupuncture for migraine prophylaxis: a randomized controlled trial. *Canadian Medical Association Journal*.

[3] May B. H., Lu C., Bennett L., Hügel H. M., Xue C. C. L. (2012). Evaluating the traditional Chinese literature for herbal formulae and individual herbs used for age-related dementia and memory impairment. *Biogerontology*.

[4] Zhang L., Li Y., Guo X. (2014). Text mining of the classical medical literature for medicines that show potential in diabetic nephropathy. *Evidence-Based Complementary and Alternative Medicine*.

[5] Olsson Y., Brun A., Englund E. (1996). Fundamental pathological lesions in vascular dementia. *Acta Neurologica Scandinavica*.

[6] Davis S., Shanahan M., Campbell A., Hegarty M., McCarthy B. (2011). Palliative care for people with dementia: aged care staff perspectives. *Alzheimer's & Dementia*.

[7] Jia J., Wang F., Wei C. (2014). The prevalence of dementia in urban and rural areas of China. *Alzheimer's and Dementia*.

[8] Ferri C. P., Prince M., Brayne C. (2005). Global prevalence of dementia: a Delphi consensus study. *The Lancet*.

[9] Quentin W., Riedel-Heller S. G., Luppa M., Rudolph A., König H.-H. (2010). Cost-of-illness studies of dementia: a systematic review focusing on stage dependency of costs. *Acta Psychiatrica Scandinavica*.

[10] Yu J., Zhang X., Liu C., Meng Y., Han J. (2006). Effect of acupuncture treatment on vascular dementia. *Neurological Research*.

[11] Shi G.-X., Liu C.-Z., Li Q.-Q., Zhu H., Wang L.-P. (2012). Influence of acupuncture on cognitive function and markers of oxidative DNA damage in patients with vascular dementia. *Journal of Traditional Chinese Medicine*.

[12] Zhong X.-Y., Su X.-X., Liu J., Zhu G. Q. (2009). Clinical effects of acupuncture combined with nimodipine for treatment of vascular dementia in 30 cases. *Journal of Traditional Chinese Medicine*.

[13] Wang T., Liu C.-Z., Yu J.-C., Jiang W., Han J.-X. (2009). Acupuncture protected cerebral multi-infarction rats from memory impairment by regulating the expression of apoptosis related genes Bcl-2 and Bax in hippocampus. *Physiology and Behavior*.

[14] Hwang I. K., Chung J. Y., Yoo D. Y. (2010). Comparing the effects of acupuncture and electroacupuncture at Zusanli and Baihui on cell proliferation and neuroblast differentiation in the rat hippocampus. *Journal of Veterinary Medical Science*.

[15] Hwang I. K., Chung J. Y., Yoo D. Y. (2010). Effects of electroacupuncture at Zusanli and Baihui on brain-derived neurotrophic factor and cyclic AMP response element-binding protein in the hippocampal dentate gyrus. *Journal of Veterinary Medical Science*.

[16] Luo Y. F., Wu J. M. (2008). *Fundamentals of Acupuncture*.

[17] Agrawal R., Srikant R. Fast algorithms for mining association rules.

[18] Agrawal R., Imielinski T., Swami A. (1993). Mining association rules between sets of items in large databases. *ACM SIGMOD Record*.

[19] Clauset A., Newman M. E. J., Moore C. (2004). Finding community structure in very large networks. *Physical Review E*.

[20] de Arruda G. F., Barbieri A. L., Rodriguez P. M. (2014). Role of centrality for the identification of influential spreaders in complex networks. *Physical Review E: Statistical, Nonlinear, and Soft Matter Physics*.

[21] Opsahl T., Agneessens F., Skvoretz J. (2010). Node centrality in weighted networks: generalizing degree and shortest paths. *Social Networks*.

[22] Brandes U. (2008). On variants of shortest-path betweenness centrality and their generic computation. *Social Networks*.

[23] Li H. Q., Li J. H., Liu A. J., Ye M. Y., Zheng G. Q. (2014). GV20-based acupuncture for animal models of acute intracerebral haemorrhage: a preclinical systematic review and meta-analysis. *Acupuncture in Medicine*.

[24] Oda E., Ohki R., Murasawa H. (2000). Noxa, a BH3-only member of the Bcl-2 family and candidate mediator of p53-induced apoptosis. *Science*.

[25] Endo H., Kamada H., Nito C., Nishi T., Chan P. H. (2006). Mitochondrial translocation of p53 mediates release of cytochrome c and hippocampal CA1 neuronal death after transient global cerebral ischemia in rats. *Journal of Neuroscience*.

[26] Zhu Y., Zeng Y. (2011). Electroacupuncture protected pyramidal cells in hippocampal CA1 region of vascular dementia rats by inhibiting the expression of P53 and Noxa. *CNS Neuroscience and Therapeutics*.

[27] Tyler W. J., Alonso M., Bramham C. R., Pozzo-Miller L. D. (2002). From acquisition to consolidation: on the role of brain-derived neurotrophic factor signaling in hippocampal-dependent learning. *Learning and Memory*.

[28] Bekinschtein P., Cammarota M., Katche C. (2008). BDNF is essential to promote persistence of long-term memory storage. *Proceedings of the National Academy of Sciences of the United States of America*.

[29] Kitagawa K. (2007). CREB and cAMP response element-mediated gene expression in the ischemic brain. *FEBS Journal*.

[30] Lee B., Sur B., Shim J., Hahm D., Lee H. (2014). Acupuncture stimulation improves scopolamine-induced cognitive impairment via activation of cholinergic system and regulation of BDNF and CREB expressions in rats. *BMC Complementary and Alternative Medicine*.

[31] Hasselmo M. E. (2006). The role of acetylcholine in learning and memory. *Current Opinion in Neurobiology*.

[32] Jay T. M. (2003). Dopamine: a potential substrate for synaptic plasticity and memory mechanisms. *Progress in Neurobiology*.

[33] Chuang C.-M., Hsieh C.-L., Li T.-C., Lin J.-G. (2007). Acupuncture stimulation at Baihui acupoint reduced cerebral infarct and increased dopamine levels in chronic cerebral hypoperfusion and ischemia-reperfusion injured Sprague-Dawley rats. *American Journal of Chinese Medicine*.

[34] Jiao H., Wang Z., Liu Y., Wang P., Xue Y. (2011). Specific role of tight junction proteins claudin-5, occludin, and ZO-1 of the blood-brain barrier in a focal cerebral ischemic insult. *Journal of Molecular Neuroscience*.

[35] Zhang Y.-M., Xu H., Sun H., Chen S., Wang F. (2014). Electroacupuncture treatment improves neurological function associated with regulation of tight junction proteins in rats with cerebral ischemia reperfusion injury. *Evidence-Based Complementary and Alternative Medicine*.

[36] Jin B.-X., Lai X.-S., Tang C.-Z. (2008). Progress in researches on the specificity of acupoints. *Acupuncture Research*.

[37] Li Y., Liang F., Yang X. (2009). Acupuncture for treating acute attacks of migraine: a randomized controlled trial. *Headache*.

[38] Ma T. T., Yu S. Y., Li Y. (2012). Randomised clinical trial: an assessment of acupuncture on specific meridian or specific acupoint vs. sham acupuncture for treating functional dyspepsia. *Alimentary Pharmacology and Therapeutics*.

[39] Shen P.-F., Kong L., Ni L.-W. (2012). Acupuncture intervention in ischemic stroke: a randomized controlled prospective study. *The American Journal of Chinese Medicine*.

[40] Huang Y., Win M. H., Chen J. (2005). A comparative study on the treatment of vascular dementia by puncturing Baihui (GV20), Shuigou (GV26) and Shenmen (HT7). *World Journal of Acupuncture—Moxibustion*.

[41] Huang Y., Lai X.-S., Tang A.-W. (2007). Comparative study of the specificities of needling acupoints DU20, DU26 and HT7 in intervening vascular dementia in different areas in the brain on the basis of scale assessment and cerebral functional imaging. *Chinese Journal of Integrative Medicine*.

[42] Alizadeh R., Esmaeili S., Shoar S., Bagheri-Hariri S., Shoar N. (2014). Acupuncture in preventing postoperative nausea and vomiting: efficacy of two acupuncture points versus a single one. *Journal of Acupuncture and Meridian Studies*.

[43] Xu F. M., Chen R. X. (2001). The antagonistic effect of acupoints on oxygen consumption rate in rats. *Journal of Jiangxi College of Traditional Chinese Medicine*.

[44] Xu F. M., Chen R. X. (2000). Effect of electroacupuncture on gastrointestinal movement in rats with hypoactive gastrointestinal movement caused by grease. *Henan Traditional Chinese Medicine*.

[45] Li X. H., Gao X. Z., Hu L. (2002). Experimental study on synergetic and antagonistic effects of acupoints Neiguan, Shenmen and Xinshu. *Chinese Acupuncture and Moxibustion*.

[46] Li X. H., Wang Q. Y., Gao X. Z. (2002). Study on cooperation and antagonism among acupoints. *Chinese Acupuncture and Moxibustion*.

[47] Feng Y., Qiu Y., Zhou X., Wang Y., Xu H., Liu B. (2013). Optimizing prescription of Chinese herbal medicine for unstable angina based on partially observable Markov decision process. *Evidence-based Complementary and Alternative Medicine*.

